# 765 When All Hell Breaks Loose: The Need for Specialized Burn Provider Expertise During Crisis

**DOI:** 10.1093/jbcr/irad045.240

**Published:** 2023-05-15

**Authors:** Anna Goff, Marcie Lambrix, Andrew Neading, Casey Kohler, Patrick Maluso, Monica Gerrek

**Affiliations:** Case Western Reserve University, MetroHealth System, Cleveland, Ohio; Case Western Reserve University, MetroHealth System, Cleveland, Ohio; MetroHealth, Macedonia, Ohio; MetroHealth Medical Center, Cleveland, Ohio; MetroHealth Medical Center, Cleveland, Ohio; MetroHealth System/Case Western Reserve University, Cleveland, Ohio; Case Western Reserve University, MetroHealth System, Cleveland, Ohio; Case Western Reserve University, MetroHealth System, Cleveland, Ohio; MetroHealth, Macedonia, Ohio; MetroHealth Medical Center, Cleveland, Ohio; MetroHealth Medical Center, Cleveland, Ohio; MetroHealth System/Case Western Reserve University, Cleveland, Ohio; Case Western Reserve University, MetroHealth System, Cleveland, Ohio; Case Western Reserve University, MetroHealth System, Cleveland, Ohio; MetroHealth, Macedonia, Ohio; MetroHealth Medical Center, Cleveland, Ohio; MetroHealth Medical Center, Cleveland, Ohio; MetroHealth System/Case Western Reserve University, Cleveland, Ohio; Case Western Reserve University, MetroHealth System, Cleveland, Ohio; Case Western Reserve University, MetroHealth System, Cleveland, Ohio; MetroHealth, Macedonia, Ohio; MetroHealth Medical Center, Cleveland, Ohio; MetroHealth Medical Center, Cleveland, Ohio; MetroHealth System/Case Western Reserve University, Cleveland, Ohio; Case Western Reserve University, MetroHealth System, Cleveland, Ohio; Case Western Reserve University, MetroHealth System, Cleveland, Ohio; MetroHealth, Macedonia, Ohio; MetroHealth Medical Center, Cleveland, Ohio; MetroHealth Medical Center, Cleveland, Ohio; MetroHealth System/Case Western Reserve University, Cleveland, Ohio; Case Western Reserve University, MetroHealth System, Cleveland, Ohio; Case Western Reserve University, MetroHealth System, Cleveland, Ohio; MetroHealth, Macedonia, Ohio; MetroHealth Medical Center, Cleveland, Ohio; MetroHealth Medical Center, Cleveland, Ohio; MetroHealth System/Case Western Reserve University, Cleveland, Ohio

## Abstract

**Introduction:**

The COVID-19 pandemic prompted health care organizations to create or revisit crisis standards of care (CSC) guidelines. The goal of these plans is to preemptively determine the most effective way to allocate resources under circumstances in which not all patients can be treated in accordance with general standards of care. In 2020, the American Burn Association (ABA) released professional recommendations for the development of CSC guidelines. Specifically, the ABA provided triage tables that could be incorporated into organizational CSC guidelines with the intention of assisting providers by predicting mortality based on a patient’s age and burn size. These tables are often misinterpreted by non-burn providers who tend to overestimate burn size and conclude that a patient has a higher predicted mortality than they actually do. Inaccurate predicted mortalities can result in inappropriate care plans and ultimately impact patient outcomes. We argue that the ABA predicted mortality tables are helpful when used by experienced burn providers, but that there are inherent ethical risks when they are utilized by providers who lack appropriate burn care experience.

**Methods:**

We conducted a literature review of CSC guidelines that included specific instructions for the triage of burn patients. We reviewed 15 publicly available CSC guidelines, 8 of which specifically mentioned burn patients, and examined each to see if/how they utilized ABA triage tables for predicting burn patient mortality.

**Results:**

Of the 8 CSC plans that acknowledged burn patients, 5 utilized the mortality predictors outlined in the ABA triage tables to guide resource allocation. In the majority of plans, patients with a predicted mortality of >90% were not eligible to receive treatment and/or transfer. None of the plans specified the involvement of a specialized burn provider in assessing burns.

**Conclusions:**

Our findings indicate that the expert evaluation of burns and thus predicted mortality can be the difference between receiving and not receiving potentially life-saving interventions under CSC guidelines. If a non-burn provider overestimates a burn size, for example, a patient may be undertreated for potentially survivable injuries under some CSC guidelines. This may further perpetuate health disparities given vulnerable populations are at higher risk of suffering burn injuries and often face a higher risk of morbidity and mortality.

**Applicability of Research to Practice:**

This project demonstrates the need for expert input from specialized burn providers in assessing burns, especially in CSC scenarios, to ensure these assessments are reliable and accurate. Involving expert burn providers in this process will help ensure ethically appropriate clinical care decisions are being made which will improve patient outcomes and resource management in crises and decrease provider distress.

